# Supplementary material for: Anthocyanin Accumulation in Grape Berry Skin Promoted by Endophytic Microbacterium sp. che218 Isolated from Wine Grape Shoot Xylem
Source: Microorganisms. 2024 Sep 19;12(9):1906. doi: 10.3390/microorganisms12091906 (PMC11434312; doi:10.3390/microorganisms12091906)
Supplement: Supplementary file 1 [file microorganisms-12-01906-s001.zip › microorganisms-3123506-supplementary.pdf]

Table S1.

Average temperatures, maximum and minimum temperatures, GDD, and precipitation from April 1 through October 31 in the experimental field

Month	Average temperature (°C)		Maximum temperature (°C)		Minimum temperature (°C)		GDD[a]		Precipitation (mm)	
	2022	2023	2022	2023	2022	2023	2022	2023	2022	2023
April	15.6	15.6	21.8	22.6	10.5	9.8	178.9	166.6	158.5	70.0
May	18.8	18.9	25.2	25.4	13.5	13.5	273.4	275.6	83.5	73.0
June	23.2	22.8	29.4	27.9	18.8	19.0	397.2	382.8	55.5	256.0
July	26.9	27.8	32.1	34.2	23.2	23.5	523.8	551.8	113.5	88.5
August	27.5	28.2	33.0	34.5	23.9	24.3	543.1	565.0	110.5	113.5
September	24.7	26.7	30.2	32.4	21.0	22.6	439.8	500.6	233	60.5
October	16.2	17.2	21.7	23.8	11.5	11.8	191.4	221.8	77	68.5
Total							2547.6	2664.2	831.5	730.0

[a] Growing degree days, base threshold of 10 °C

Table S2.

Isolates used in phylogenetic analysis of 16S rDNA nucleotide sequences

species	isolate	accession number
Microbacterium enclense	bin7	CP116226
Microbacterium sediminis	YLB-01	CP038256
Microbacterium lemovicicum	Viu22	CP031423
Microbacterium binotii	Au-Mic3	CP090347
Microbacterium testaceum	D12	CP103793
Microbacterium testaceum	StLB037	AP012052
Microbacterium testaceum	HW4	NZ_CP121699
Microbacterium hominis	01094	CP061345
Microbacterium hominis	PDNC016	CP070346
Microbacterium hominis	PA2F3	CP054038
Microbacterium proteolyticum	ustc	NZ_CP121274
Microbacterium kitamiense kitami	C2	NR_112042
Microbacterium chocolatum	SIT 101	CP015810
Microbacterium aurum	KACC 15219	CP018762
Microbacterium paulum	2C	NR_181679
Microbacterium schleiferi	A32-1	CP064760
Microbacterium oleivorans	I46	CP058316
Microbacterium terricola	JCM 14903	CP107222
Microbacterium terricola	KV-448	AP027141
Microbacterium caowuchunii	ST-M6	CP044231
Microbacterium wangchenii	dk512	CP038266
Microbacterium invictum	X-18	CP139779
Microbacterium rhizosphaerae	KEMB	CP139368
Microbacterium paludicola	CC3	CP018134
Microbacterium arborescens	SSF12	CP128474
Microbacterium elymi	KUDC0405	CP091139
Microbacterium oxydans	HG3	CP031422
Microbacterium oxydans	VIU2A	CP031338
Microbacterium liquefaciens	DND4	CP101471
Microbacterium oxydans	ML-6	CP092891
Microbacterium liquefaciens	MWF001	CP130429
Microbacterium liquefaciens	B1075	CP118606
Microbacterium foliorum	NRRL B-24224	CP031425
Microbacterium foliorum	M2	CP041040
Microbacterium oxydans	B5	DQ350825
Microbacterium paraoxydans	70447	CP064873
Microbacterium azadirachtae	CNUC13	CP099706
Microbacterium resistens	MZT7	CP082781

Table S2.

continued

Microbacterium neungamense	EF45044	CP069717
Microbacterium esteraromaticum	S29	AB099658
Microbacterium esteraromaticum	DEHP-1	CP118100
Okibacterium endophyticum	EGI 650022	NR_136851.2
Leucobacter insecticola	HDW9B	CP049934
Curtobacterium flaccumfaciens	Sample7_18	CP121450
Curtobacterium citreum	A02	CP126586
Humibacter ginsenosidimutans	WJ7-1	CP042305
Antiquaquibacter oligotrophicus	SG_E_30_P1	CP085036
Leifsonia xyli	SE134	CP014761
Leifsonia shinshuensis	INR9	CP043641
Agromyces mangrovi	NBRC 112812	AP027737
Methylobacterium indicum	JDJ13	NZ_CP121700
Bacillus subtilis subsp. *subtilis*	str. 168	CP053102
